# Is Early Appropriate Care of axial and femoral fractures appropriate in multiply-injured elderly trauma patients?

**DOI:** 10.1186/s13018-016-0441-7

**Published:** 2016-09-26

**Authors:** M. S. Reich, A. J. Dolenc, T. A. Moore, H. A. Vallier

**Affiliations:** Department of Orthopaedic Surgery, Case Western Reserve University, The MetroHealth System, 2500 MetroHealth Drive, Cleveland, OH 44109 USA

**Keywords:** Early Appropriate Care, Acidosis, Base excess, Fixation timing, Resuscitation, Elderly polytrauma

## Abstract

**Background:**

Previous work established resuscitation parameters that minimize complications with early fracture management. This Early Appropriate Care (EAC) protocol was applied to patients with advanced age to determine if they require unique parameters to mitigate complications.

**Methods:**

Between October 2010 and March 2013, 376 consecutive skeletally mature patients with unstable fractures of the pelvis, acetabulum, thoracolumbar spine, and/or proximal or diaphyseal femur fractures were treated at a level I trauma center and were prospectively studied. Patients aged ≤30 years (*n* = 114), 30 to 60 years (*n* = 184), and ≥60 years (*n* = 37) with Injury Severity Scores (ISS) ≥16 and unstable fractures of the pelvis, acetabulum, spine, and/or diaphyseal femur were treated within 36 h, provided they showed evidence of adequate resuscitation. ISS, Glasgow Coma Scale (GCS), and American Society of Anesthesiologists (ASA) classification were determined. Lactate, pH, and base excess (BE) were measured at 8-h intervals. Complications included pneumonia, pulmonary embolism (PE), acute renal failure, acute respiratory distress syndrome (ARDS), multiple organ failure (MOF), deep vein thrombosis, infection, sepsis, and death.

**Results:**

Patients ≤30 years old (y/o) were more likely to sustain gunshot wounds (*p* = 0.039), while those ≥60 y/o were more likely to fall from a height (*p* = 0.002). Complications occurred at similar rates for patients ≤30 y/o, 30 to 60 y/o, and ≥60 y/o. There were no differences in lactate, pH, or BE at the time of surgery. For patients ≤30 y/o, there were increased overall complications if pH was <7.30 (*p* = 0.042) or BE <−6.0 (*p* = 0.049); patients ≥60 y/o demonstrated more sepsis if BE was <−6.0 (*p* = 0.046).

**Conclusions:**

EAC aims to definitively manage axial and femoral shaft fractures once patients have been adequately resuscitated to minimize complications. EAC is associated with comparable complication rates in young and elderly patients. Further study is warranted with a larger sample to further validate EAC in elderly patients. Level of evidence: level II prospective, comparative study.

## Background

Controversy persists with respect to the decision for early total care (ETC) versus damage-control orthopedics (DCO). Mechanically unstable fractures of the femur, pelvis, acetabulum, and thoracolumbar spine often require bedrest until surgical intervention. Early stabilization has been advocated by many authors with regard to fractures of the femur [1–5], pelvis [6–9], acetabulum [8, 9], and spine [10–16], allowing for expeditious mobilization. This is associated with reduced complication rates in resuscitated patients, particularly pulmonary issues and sepsis [2–5, 10–12]. These effects may be more pronounced in the setting of polytrauma [1, 3, 17, 18].

Conversely, the “second-hit” theory suggests that the insult of early definitive surgery, combined with that of the initial trauma, may overwhelm the body with an exuberant inflammatory response, leading to more complications [19–22]. DCO proposes provisional stabilization to minimize these complications, while providing time for the body to recover [22–24]. This necessarily results in additional operations and implants and longer hospital stays. Some fracture patterns, such as those of the acetabulum, pelvis, and cervical or thoracolumbar spine may preclude DCO and, even if provisional stabilization is possible, may not permit early mobilization.

There are likely optimal conditions along the spectrum from ETC to DCO when early care is appropriate and may be undertaken. Major trauma often requires profound resuscitative efforts to correct blood and volume losses and the ensuing metabolic acidosis, reflected by changes in pH and base excess [25]. This necessitates transfusions of crystalloid, colloid, packed red blood cells (PRBC), fresh frozen plasma (FFP), and platelets (Plt). Metabolic acidosis on presentation is a prognostic indicator for the development of pulmonary complications [18, 26, 27], organ dysfunction [27, 28], and death [27–30].

Prior work from our institution led to the development of parameters designed to minimize complications. Definitive management of mechanically unstable fractures of the femur, pelvis, acetabulum, and thoracolumbar spine was performed within 36 h in patients who demonstrated a positive response to resuscitative efforts, defined as having either a lactate <4.0 mmol/L, pH ≥7.25, or base excess (BE) ≥−5.5 mmol/L [18, 31]. In the current study, we have applied this protocol prospectively, with the purpose of assessing its safety in older patients. We sought to determine if patients with advanced age require unique parameters to mitigate the risk of complications and mortality. Thus, we hypothesized that with adequate resuscitation, there would be no differences in complication rates between younger and older multiply-injured trauma patients.

## Methods

This study was approved by the MetroHealth System Institutional Review Board (study number IRB07-01157). Between October 2010 and March 2013, a total of 376 consecutive patients were treated at a level I trauma center. Early Appropriate Care (EAC) was implemented in 2010, and relevant data were prospectively collected. All patients had an Injury Severity Score (ISS) ≥16 and were either skeletally mature or were approaching skeletal maturity and presented with fracture patterns requiring the standard fixation methods used in adults. There were 139 patients ≤30 years old (y/o), 184 patients 30 to 60 y/o, and 52 patients ≥60 y/o. All had achieved the desired level of resuscitation as measured by the correction of acidosis within 36 h after injury. Mechanisms of injury were categorized as falls from greater than 6 ft of height, motor vehicle collisions, motorcycle collisions, gunshot wounds (GSW), pedestrian versus automotive injuries, combination injuries, and other. Low-energy fractures were excluded. The time from injury until definitive fixation was determined. When patients had multiple unstable fractures, the timing of the final treated fracture was used for data analysis. Fractures that could be readily temporized with splinting (e.g., ankle and forearm fractures) were not included. The timing and techniques of fracture fixation were recorded.

As previously described [18], associated injuries were documented and their severity was assessed using the ISS [32], Abbreviated Injury Scale (AIS), and Glasgow Coma Scale (GCS) [33]. The American Society of Anesthesiologists (ASA) score was recorded. Laboratory studies including pH, BE, and lactate were documented every 8 h upon presentation to the emergency department and perioperatively. If a patient demonstrated sufficient resuscitation, the assessment of pH, BE, and lactate was discontinued, unless otherwise indicated by the trauma service. Patients were determined to be adequately resuscitated if they demonstrated one of the following: either lactate <4.0 mmol/L, pH ≥7.25, or BE ≥−5.5 mmol/L [18, 31]. For data analysis, the values closest to the time of surgery were used, as these were the most representative of resuscitation status at the time of definitive fixation. Transfusion requirements, ventilator use, length of stay (LOS) in the intensive care unit (ICU), and length of hospitalization were documented with durations recorded to the nearest full day. Complications were recorded, including wound infection, pulmonary complications (acute respiratory distress syndrome (ARDS), pneumonia, pulmonary embolism (PE)), acute renal failure (ARF), multiple organ failure (MOF), and deep venous thrombosis (DVT). All patients were followed for a minimum of 6 months after injury to assess for complications and readmissions.

Statistical analysis was performed using Microsoft Excel (Microsoft, Redmond, WA) and GraphPad (GraphPad Software, La Jolla, CA). *T* tests, Fisher exact tests, ANOVA, and Mann-Whitney *U* tests were performed with parametric tests used if the data was normally distributed and non-parametric tests if the data was not normally distributed. Statistical significance was set at *p* < 0.05.

## Results

One hundred fourteen patients ≤30 y/o, 184 patients 30 to 60 y/o, and 37 patients ≥60 y/o were studied. Younger patients (mean ± standard deviation (SD) 23.2 ± 4.1 y/o, range 16–30 y/o) were significantly younger than the older patients (mean ± SD 68.7 ± 9.0 y/o, range 60–91 y/o, *p* < 0.001). Younger patients had lower GCS (mean ± SD 12.3 ± 4.3 versus 14.2 ± 2.8, *p* = 0.003) and ASA (mean ± SD 2.58 ± 0.86 versus 3.03 ± 0.76, *p* = 0.002) with no difference in ISS (25.0 ± 9.6 versus 24.6 ± 9.0) when compared with the oldest patients. No differences in GCS, ASA, or ISS were noted when the middle age group was compared with other groups. Younger patients were more likely to sustain GSWs (*p* = 0.039), while older patients were more likely to have sustained falls (*p* = 0.002). Injury mechanisms are summarized in Table 1.

**Table 1:** Mechanisms of injuries. Patients in the younger and older age groups are compared

	≤30 y/o (*n* = 114)	≥60 y/o (*n* = 37)	*p*
Falls	17 (14.9 %)	15 (40.5 %)	0.002
MVC	60 (52.6 %)	18 (48.6 %)	0.708
MCC	14 (12.3 %)	4 (10.8 %)	1.000
GSW	12 (10.5 %)	0 (0.0 %)	0.039
Pedestrian	7 (6.1 %)	0 (0.0 %)	0.195
Combo	3 (2.6 %)	0 (0.0 %)	1.000
Other	1 (0.9 %)	0 (0.0 %)	1.000

*MVC* motor vehicle collision, *MCC* motorcycle collision, *GSW* gunshot wound

Patients ≤30 y/o sustained 125 mechanically unstable fractures including 70 femur fractures (four patients had bilateral femur fractures), 15 pelvic fractures, 10 acetabulum fractures, and 30 thoracolumbar spine fractures treated surgically. Patients 30 to 60 y/o sustained 84 femur fractures, 51 pelvis fractures, 40 acetabulum fractures, and 36 thoracolumbar spine fractures treated surgically. Patients ≥60 y/o had 19 femur fractures (three patients had bilateral femur fractures), 5 pelvic fractures, 7 acetabulum fractures, and 13 thoracolumbar spine fractures treated surgically. Elderly patients trended toward more acetabulum fractures (*p* = 0.085), with no differences in frequency of femur, pelvic, or spine fractures. Fracture patterns are summarized in Tables 2 and 3.

**Table 2:** AO/OTA classification for femur fractures

Classification	≤30 y/o (*N* ^a^)	≥60 y/o (*N*)
31A	5	8
31B	5	2
31C	0	0
32A	22	3
32B	26	3
32C	12	3

^a^4 patients ≤30 y/o had ipsilateral femur fractures

**Table 3:** AO/OTA classification for pelvis and acetabulum fractures

Classification	≤30 y/o (*N*)	≥60 y/o (*N*)
61A	0	0
61B	5	2
61C	10	3
62A	5	2
62B	4	3
62C	1	2

Associated injuries are included in Table 4. Elderly patients were less likely to have head injuries (*p* = 0.048) when compared with any patients less than age 60 years, but there were no differences in frequency or severity of abdominal or chest injuries among the three different age groups. The presence and severity of associated injuries among the younger and older age groups are shown in the table. Transfusion requirements are listed in Table 5. Elderly patients trended toward more FFP transfusions (*p* = 0.082) and required more cryoprecipitate transfusions (*p* = 0.007) versus patients in the youngest group.

**Table 4:** Associated injuries

	≤30 y/o (*n* = 114)	≥60 y/o (*n* = 37)	*p*
Abdominal injury			
Any	31 (27.2 %)	7 (18.9 %)	0.387
Minor	19 (16.7 %)	3 (8.1 %)	0.285
Severe	12 (10.5 %)	4 (10.8 %)	1.000
Chest injury			
Any	62 (54.4 %)	19 (51.4 %)	0.850
Minor	28 (24.6 %)	9 (24.3 %)	1.000
Severe	34 (29.8 %)	10 (27.0 %)	0.837
Head injury			
Any	78 (68.4 %)	18 (48.6 %)	0.048
Minor	54 (47.4 %)	12 (32.4 %)	0.130
Severe	24 (21.1 %)	6 (16.2 %)	0.639

Minor injuries to the chest and abdomen had AIS of 1 or 2, while severe injuries had AIS of 3 or higher. Minor head injuries had presenting GCS >8, while severe injuries had lower GCS

**Table 5:** The number of patients having transfusion of one or more blood products is listed

	≤30 y/o (*n* = 114)	30 to 60 y/o (*n* = 184)	≥60 y/o (*n* = 37)	*p*
PRBC	66 (57.9 %)	110 (60 %)	26 (70.3 %)	0.245
FFP	16 (14.0 %)	32 (17.4 %)	10 (27.0 %)	0.082
Plt	8 (7.0 %)	15 (8.2 %)	9 (24.3 %)	0.007
Cryo	2 (1.8 %)	2 (1.1 %)	1 (2.7 %)	0.573
Any	68 (59.6 %)	112 (61 %)	28 (75.7 %)	0.115

*p* values are shown for the youngest versus the oldest patients. All *p* values for the middle age group versus the other groups were nonsignificant (*p* > 0.05)

*PRBC* packed red blood cells, *FFP* fresh frozen plasma, *Plt* platelets, *Cryo* cryoprecipitate

Patients less than 30 y/o trended toward fewer ventilator days (≤30 y/o: median 0, inter-quartile range (IQR) 0–1 days, min 0 days, max 24 days; ≥60 y/o: median 0, IQR 0–3 days, min 0 days, max 26 days, *p* = 0.078). There was no significant difference between age groups in terms of ICU stay with younger patients having a median 1-day stay (IQR 0–4 days, min 0 days, max 33 days), while older patients spent a median of 3 days (IQR 0–6 days, min 0 days, max 38 days). Younger patients were admitted a median of 6 days (IQR 4–10 days, min 1 day, max 46 days), while older patients were admitted a median of 8 days (IQR 5–14 days, min 2 days, max 38 days, *p* = 0.05). No differences were noted when patients in the middle age group were compared with other age groups in terms of length of stay or ventilator days.

The incidence of complications was similar for all age cohorts (≤30 y/o 15.8 %, 30 to 60 y/o 19.5 %, versus ≥60 y/o 16.2 %). All complications are included in Table 6. Younger patients were more likely to develop a PE or ARDS (both *p* = 0.045), when compared with the oldest patients. There were no differences in the occurrence of the other complications assessed. In general, higher ASA was associated with a greater incidence of any complication, pulmonary complication, pneumonia, ARDS, MOF, sepsis, and death, irrespective of patient age.

**Table 6:** Complications for patients ≤30 y/o, 30 to 60 y/o, and ≥60 y/o

	≤30 y/o (*n* = 114)	30 to 60 y/o (*n* = 184)	≥60 y/o (*n* = 37)	*p*
Any	18 (15.8 %)	36 (19.5 %)	6 (16.2 %)	0.84
Pulmonary	14 (12.3 %)	22 (12.0 %)	3 (8.1 %)	0.46
Pneumonia	8 (7.0 %)	20 (10.9 %)	3 (8.1 %)	0.86
PE	4 (3.5 %)	4 (2.2 %)	0 (0.0 %)	0.045
ARF	0 (0.0 %)	2 (1.1 %)	1 (2.7 %)	0.32
ARDS	4 (3.5 %)	1 (0.5 %)	0 (0.0 %)	0.045
MOF	0 (0.0 %)	1 (0.5 %)	1 (2.7 %)	0.32
DVT	2 (1.8 %)	4 (2.2 %)	0 (0.0 %)	0.16
Infection	3 (2.6 %)	6 (3.3 %)	0 (0.0 %)	0.083
Sepsis	1 (0.9 %)	5 (2.7 %)	2 (5.4 %)	0.25
Death	2 (1.8 %)	2 (1.1 %)	1 (2.7 %)	0.75

*p* values are shown for the youngest versus the oldest patients. All *p* values for the middle age group versus the youngest group were nonsignificant (*p* > 0.05)

*Pulmonary* pulmonary complications include pneumonia, ARDS, and PE; *ARDS* adult respiratory distress syndrome; *PE* pulmonary embolism; *ARF* acute renal failure; *MOF* multiple organ failure; *DVT* deep venous thrombosis

At the time of fixation, for patients ≤30 y/o, 30 to 60 y/o, and ≥60 y/o, there were no differences in lactate (2.09 ± 0.95 mmol/L vs. 2.03 ± 0.82 mmol/L vs. 1.86 ± 0.81 mmol/L, respectively), pH (7.32 ± 0.071 vs. 7.31 ± 0.069 vs. 7.32 ± 0.088, respectively), or BE (−3.79 ± 3.7 mmol/L vs. −3.64 ± 3.8 mmol/L vs. −3.42 ± 4.3 mmol/L, respectively). Subgroup analysis evaluating the severity of acidosis incrementally within patients ≤30 y/o showed increased overall complications if pH was <7.30 (*p* = 0.042) or BE <−6.0 mmol/L (*p* = 0.049). There were trends toward more overall complications with BE <−5.5 mmol/L (*p* = 0.058), pulmonary complications with BE <−6.0 mmol/L (*p* = 0.059), and more pneumonia with pH <7.2 (*p* = 0.061) or <7.25 (*p* = 0.080). Patients ≥60 y/o demonstrated more sepsis if BE was <−6.0 mmol/L (*p* = 0.046) and trended toward more sepsis with BE <−5.5 mmol/L or <−5.0 mmol/L (both *p* = 0.086). There were trends toward more overall complications with BE <−6.0 mmol/L (*p* = 0.062) and increased MOF with pH <7.2 (*p* = 0.077) and death with pH <7.2 (*p* = 0.077).

## Discussion

The goal of EAC is to definitively stabilize fractures of the femur, pelvis, acetabulum, and spine after adequate resuscitation, such that the risks of complications are minimized. Patients who are hemodynamically stable and are demonstrating objective evidence of adequate resuscitation may benefit from early fixation. The advantages of early fixation of unstable axial and femoral fractures appear to outweigh the risks of complications in patients with a lactate <4.0 mmol/L, pH ≥7.25, or BE ≥−5.5 mmol/L [18, 31, 34, 35]. These parameters were initially developed and tested in skeletally mature patients of all ages. In this study, we used these parameters to compare the results of treating an elderly patient population with high-energy injuries and multiple system involvement.

Elderly patients represent a unique population of great importance as this cohort of patients continues to grow. The rate of mortality is substantially higher in the elderly population compared to younger patients [36]. Mortality was found to increase 6.8 % per year in elderly trauma patients over 65 y/o [37], and pre-existing medical conditions place patients in this age group at greater risk [37, 38]. ISS, GCS, emergent intubation, coagulopathy, anemia, fluid requirements, and pre-existing chronic renal failure have been shown to be predictors of mortality [39–41].

Resuscitation is paramount to treating the elderly population, a population that may require more intensive resuscitation and monitoring [42–44]. However, it must be undertaken carefully, as high-volume resuscitation was shown to be an independent predictor of mortality in patients ≥70 y/o [45]. In the elderly population, measures of metabolic acidosis including abnormal lactate and base deficit have been linked to increased mortality [29, 46]. Davis and Kaups compared patients ≥55 y/o against younger patients and found greater mortality for older patients with base deficits on presentation [47].

The timing of definitive fixation must also be given consideration. In elderly patients with hip fractures, mortality increased if surgery was delayed more than 3 days [48, 49] or 4 days [50]. Kenzora et al. found that patients operated on within 24 h had a higher mortality than those delayed 2 to 5 days and that patients with more comorbidities had a higher mortality. The authors recommended resuscitating the patients and treating active medical issues prior to surgical intervention [51]. In contrast, a multi-center study of trauma patients ≥60 y/o, excluding “slip-and-fall” injuries, showed that patients who required only orthopedic surgery, including early fixation of long-bone fractures and external fixation of pelvic fractures, were less likely to die than those requiring no surgery. Timing, ≤24 h or >24 h, was not found to be a risk factor for mortality [39].

In the current study, in the context of the resuscitation parameters, there were no differences between ≤30 y/o, age 30 to 60 y/o, and patients ≥60 y/o with respect to lactate, pH, or BE at the time of definitive fixation. This is consistent with all patients being similarly resuscitated according to the same guidelines. It is reassuring that when using the EAC protocol, the rate of complications was similar in patients ≤30 y/o and patients ≥60 y/o at 15.8 and 16.2 %, respectively. Moreover, all groups met the goal of fewer than 20 % overall pulmonary complications (12.3 and 8.1 %, respectively, for the younger and older cohorts) [18]. These data suggest that, independent of age, markers of resuscitation may dictate the appropriate timing of surgery. Nevertheless, in the younger cohort, patients were more likely to have complications with a lower pH or BE, and older patients demonstrated more sepsis with a lower BE. Therefore, there may still be age-specific risks associated with acidosis on presentation.

Our study is strengthened by its prospective nature. As such, we were able to employ a standardized protocol in treating all patients. This is in addition to the standardization at our institution for the administration of perioperative antibiotics and DVT prophylaxis. The regimen for obtaining laboratory values was consistent and accurately reflected the resuscitation status at the time of definitive fixation. However, our study is limited in that the frequency and timing of non-orthopedic procedures was not recorded. The protocol is meant to be generalizable to the elderly trauma population, but we recognize not all fractures behave similarly, and future study into variations in the protocol for specific fractures or other injuries and baseline morbidities is warranted. Determining which lab values, or which combinations of lab values, are most predictive of patient outcomes would also be valuable. Further, with relatively small groups and low incidences of complications in this study, larger groups of patients will be necessary for more statistically and clinically significant conclusions to be made.

## Conclusions

In conclusion, the Early Appropriate Care of elderly patients with high-energy mechanism and multiple trauma appears to be a viable treatment algorithm. While historically the rates of complications in the elderly population have exceeded that of their younger counterparts, in the current study, although groups were small, there appears to be comparable complication rates and hospital course in adequately resuscitated patients, as measured by the correction of metabolic acidosis, irrespective of age. Further study is certainly indicated to expand on these results, to evaluate the impact of pre-existing medical conditions and ASA scores, and to study a larger cohort of patients.

## Abbreviations

ARDS: Acute respiratory distress syndrome
ASA: American Society of Anesthesiologists
BE: Base excess
DCO: Damage-control orthopedics
EAC: Early Appropriate Care
ETC: Early total care
GCS: Glasgow Coma Scale
ISS: Injury Severity Score
MOF: Multiple organ failure
PE: Pulmonary embolism

## References

[1] Bone LB, Johnson KD, Weigelt J, Scheinberg R (1989). Early versus delayed stabilization of femoral fractures. A prospective randomized study. J Bone Joint Surg Am.

[2] Brundage SI, McGhan R, Jurkovich GJ, Mack CD, Maier RV (2002). Timing of femur fracture fixation: effect on outcome in patients with thoracic and head injuries. J Trauma.

[3] Lefaivre KA, Starr AJ, Stahel PF, Elliott AC, Smith WR (2010). Prediction of pulmonary morbidity and mortality in patients with femur fracture. J Trauma.

[4] Nahm NJ, Como JJ, Wilber JH, Vallier HA (2011). Early appropriate care: definitive stabilization of femoral fractures within 24 hours of injury is safe in most patients with multiple injuries. J Trauma.

[5] Charash WE, Fabian TC, Croce MA (1994). Delayed surgical fixation of femur fractures is a risk factor for pulmonary failure independent of thoracic trauma. J Trauma.

[6] Latenser BA, Gentilello LM, Tarver AA, Thalgott JS, Batdorf JW (1991). Improved outcome with early fixation of skeletally unstable pelvic fractures. J Trauma.

[7] Riemer BL, Butterfield SL, Diamond DL, Young JC, Raves JJ, Cottington E (1993). Acute mortality associated with injuries to the pelvic ring: the role of early patient mobilization and external fixation. J Trauma.

[8] Vallier HA, Cureton BA, Ekstein C, Oldenburg FP, Wilber JH (2010). Early definitive stabilization of unstable pelvis and acetabulum fractures reduces morbidity. J Trauma.

[9] Plaisier BR, Meldon SW, Super DM, Malangoni MA (2000). Improved outcome after early fixation of acetabular fractures. Injury.

[10] Dimar JR, Carreon LY, Riina J, Schwartz DG, Harris MB (2010). Early versus late stabilization of the spine in the polytrauma patient. Spine (Phila Pa 1976).

[11] McHenry TP, Mirza SK, Wang J, Wade CE, O’Keefe GE, Dailey AT (2006). Risk factors for respiratory failure following operative stabilization of thoracic and lumbar spine fractures. J Bone Joint Surg Am.

[12] Bliemel C, Lefering R, Buecking B, Frink M, Struewer J, Krueger A (2014). Early or delayed stabilization in severely injured patients with spinal fractures? Current surgical objectivity according to the Trauma Registry of DGU: treatment of spine injuries in polytrauma patients. J Trauma Acute Care Surg.

[13] McLain RF, Benson DR (1999). Urgent surgical stabilization of spinal fractures in polytrauma patients. Spine (Phila Pa 1976).

[14] Pakzad H, Roffey DM, Knight H, Dagenais S, Yelle JD, Wai EK (2011). Delay in operative stabilization of spine fractures in multitrauma patients without neurologic injuries: effects on outcomes. Can J Surg.

[15] O’Boynick CP, Kurd MF, Darden BV, Vaccaro AR, Fehlings MG (2014). Timing of surgery in thoracolumbar trauma: is early intervention safe?. Neurosurg Focus.

[16] Stahel PF, VanderHeiden T, Flierl MA, Matava B, Gerhardt D, Bolles G (2013). The impact of a standardized “spine damage-control” protocol for unstable thoracic and lumbar spine fractures in severely injured patients: a prospective cohort study. J Trauma Acute Care Surg.

[17] Johnson KD, Cadambi A, Seibert GB (1985). Incidence of adult respiratory distress syndrome in patients with multiple musculoskeletal injuries: effect of early operative stabilization of fractures. J Trauma.

[18] Vallier HA, Wang X, Moore TA, Wilber JH, Como JJ (2013). Timing of orthopaedic surgery in multiple trauma patients: development of a protocol for early appropriate care. J Orthop Trauma.

[19] Giannoudis PV, Smith RM, Bellamy MC, Morrison JF, Dickson RA, Guillou PJ (1999). Stimulation of the inflammatory system by reamed and unreamed nailing of femoral fractures. An analysis of the second hit. J Bone Joint Surg Br.

[20] Morley JR, Smith RM, Pape HC, MacDonald DA, Trejdosiewitz LK, Giannoudis PV (2008). Stimulation of the local femoral inflammatory response to fracture and intramedullary reaming: a preliminary study of the source of the second hit phenomenon. J Bone Joint Surg Br.

[21] Pape HC, Griensven MV, Hildebrand FF, Tzioupis CT, Sommer KL, Krettek CC (2008). Systemic inflammatory response after extremity or truncal fracture operations. J Trauma.

[22] Pape HC, Hildebrand F, Pertschy S, Zelle B, Garapati R, Grimme K (2002). Changes in the management of femoral shaft fractures in polytrauma patients: from early total care to damage control orthopedic surgery. J Trauma.

[23] O’Toole RV, O’Brien M, Scalea TM, Habashi N, Pollak AN, Turen CH (2009). Resuscitation before stabilization of femoral fractures limits acute respiratory distress syndrome in patients with multiple traumatic injuries despite low use of damage control orthopedics. J Trauma.

[24] Pape HC (2008). Effects of changing strategies of fracture fixation on immunologic changes and systemic complications after multiple trauma: damage control orthopedic surgery. J Orthop Res.

[25] Dalal SA, Burgess AR, Siegel JH, Young JW, Brumback RJ, Poka A (1989). Pelvic fracture in multiple trauma: classification by mechanism is key to pattern of organ injury, resuscitative requirements, and outcome. J Trauma.

[26] Eberhard LW, Morabito DJ, Matthay MA, Mackersie RC, Campbell AR, Marks JD (2000). Initial severity of metabolic acidosis predicts the development of acute lung injury in severely traumatized patients. Crit Care Med.

[27] Davis JW, Parks SN, Kaups KL, Gladen HE, O’Donnell-Nicol S (1996). Admission base deficit predicts transfusion requirements and risk of complications. J Trauma.

[28] Guyette F, Suffoletto B, Castillo JL, Quintero J, Callaway C, Puyana JC (2011). Prehospital serum lactate as a predictor of outcomes in trauma patients: a retrospective observational study. J Trauma.

[29] Callaway DW, Shapiro NI, Donnino MW, Baker C, Rosen CL (2009). Serum lactate and base deficit as predictors of mortality in normotensive elderly blunt trauma patients. J Trauma.

[30] Claridge JA, Crabtree TD, Pelletier SJ, Butler K, Sawyer RG, Young JS (2000). Persistent occult hypoperfusion is associated with a significant increase in infection rate and mortality in major trauma patients. J Trauma.

[31] Vallier HA, Moore TA, Como JJ, Wilczewski PA, Steinmetz MP, Wagner KG (2015). Complications are reduced with a protocol to standardize timing of fixation based on response to resuscitation. J Orthop Surg and Res.

[32] Baker SP, O’Neill B, Haddon W, Long WB (1974). The injury severity score: a method for describing patients with multiple injuries and evaluating emergency care. J Trauma.

[33] Teasdale G, Jennett B (1974). Assessment of coma and impaired consciousness. A practical scale. Lancet.

[34] Weinberg DS, Narayanan AS, Moore TA, Vallier HA (2015). Prolonged resuscitation of metabolic acidosis after trauma is associated with more complications. J Orthop Surg and Res.

[35] Vallier HA, Dolenc AJ, Moore TA. Early Appropriate Care: a protocol to standardize resuscitation assessment and to expedite fracture care reduces hospital stay and enhances revenue. J Orthop Trauma. 2016. Epub. 10.1097/BOT.0000000000000524 26741643

[36] Giannoudis PV, Harwood PJ, Court-Brown C, Pape HC (2009). Severe and multiple trauma in older patients; incidence and mortality. Injury.

[37] Grossman MD, Miller D, Scaff DW, Arcona S (2002). When is an elder old? Effect of preexisting conditions on mortality in geriatric trauma. J Trauma.

[38] Perdue PW, Watts DD, Kaufmann CR, Trask AL (1998). Differences in mortality between elderly and younger adult trauma patients: geriatric status increases risk of delayed death. J Trauma.

[39] Tornetta P, Mostafavi H, Riina J, Turen C, Reimer B, Levine R (1999). Morbidity and mortality in elderly trauma patients. J Trauma.

[40] Schoeneberg C, Probst T, Schilling M, Wegner A, Hussmann B, Lendemans S (2014). Mortality in severely injured elderly patients: a retrospective analysis of a German level 1 trauma center (2002-2011). Scand J Trauma Resusc Emerg Med.

[41] Bala M, Willner D, Klauzni D, Bholah-Abram T, Rivkind A, Gazala MA (2013). Pre-hospital and admission parameters predict in-hospital mortality among patients 60 years and older following severe trauma. Scand J Trauma Resusc Emerg Med.

[42] Switzer JA, Gammon SR (2012). High-energy skeletal trauma in the elderly. J Bone Joint Surg Am.

[43] Heffernan DS, Thakkar RK, Monaghan SF, Ravindran R, Adams CA, Kozloff MS (2010). Normal presenting vital signs are unreliable in geriatric blunt trauma victims. J Trauma.

[44] Scalea TM, Simon HM, Duncan AO, Atweh NA, Sclafani SJ, Phillips TF (1990). Geriatric blunt multiple trauma: improved survival with early invasive monitoring. J Trauma.

[45] Ley EJ, Clond MA, Srour MK, Barnajian M, Mirocha J, Margulies DR (2011). Emergency department crystalloid resuscitation of 1.5 L or more is associated with increased mortality in elderly and nonelderly trauma patients. J Trauma.

[46] Neville AL, Nemtsev D, Manasrah R, Bricker SD, Putnam BA (2011). Mortality risk stratification in elderly trauma patients based on initial arterial lactate and base deficit levels. Am Surg.

[47] Davis JW, Kaups KL (1998). Base deficit in the elderly: a marker of severe injury and death. J Trauma.

[48] Rogers FB, Shackford SR, Keller MS (1995). Early fixation reduces morbidity and mortality in elderly patients with hip fractures from low-impact falls. J Trauma.

[49] Zuckerman JD, Skovron ML, Koval KJ, Aharonoff G, Frankel VH (1995). Postoperative complications and mortality associated with operative delay in older patients who have a fracture of the hip. J Bone Joint Surg Am.

[50] Moran CG, Wenn RT, Sikand M, Taylor AM (2005). Early mortality after hip fracture: is delay before surgery important?. J Bone Joint Surg Am.

[51] Kenzora JE, McCarthy RE, Lowell JD, Sledge CB (1984). Hip fracture mortality. Relation to age, treatment, preoperative illness, time of surgery, and complications. Clin Orthop Relat Res.

